# Supplementary material for: Development of a computer-aided design software for the quantitative evaluation of aesthetic damage
Source: PLoS One. 2019 Dec 18;14(12):e0226322. doi: 10.1371/journal.pone.0226322 (PMC6919621; doi:10.1371/journal.pone.0226322)
Supplement: S1 Code site — 1. (ZIP) [file pone.0226322.s001.zip › [EESC_jr][Projeto]Relatorio_07_21/reports/templates_zip/formwizard-master.zip!/formwizard-master/examples/example_14_using_fieldset_as_step_identifier.html]

xml version="1.0" encoding="UTF-8"?


JQuery Form Wizard


### Example of a straight wizard with the steps defined using fieldset elements

- Straight wizard with three steps.
- Steps are defined using fieldset elements. One could use e.g. div elements etc as well.
- Validation plugin enabled for the email field on the second step.
- Form plugin enabled and beforeSubmit and success callbacks are used.

---


# First step - Name

  
First name  
  
Surname  
  


# Step 2 - Personal information

  
Social Security Number  


 -
  
Phone number  
 -
 -
  
\*Email  
  


# Last step - Username

  
User name  
  
Password  
  
Retype password


---
